# Reliability and validity of the Addiction-Like eating behavior scale (AEBS) in a Peruvian adolescent population

**DOI:** 10.3389/fpsyg.2025.1490893

**Published:** 2025-06-18

**Authors:** Joel Figueroa-Quiñones, Nelly T. Condor Heredia, Julio Cjuno, Daniel E. Yupanqui-Lorenzo, Walter Jesús Acharte-Champi, Khristel Fernandini-Valenzuela

**Affiliations:** ^1^Universidad Autonoma de Ica, Escuela de Psicologia, Ica, Peru; ^2^Universidad Cesar Vallejo, Escuela de Medicina, Piura, Peru; ^3^Centro de Investigación e-Health, Universidad de Ciencias y Humanidades, Lima, Peru

**Keywords:** food addiction, adolescents, eating behavior, reliability, validation

## Abstract

**Background:**

Understanding eating behaviors in Latin American contexts is crucial, given the current epidemic of obesity among adolescents in this region.

**Objective:**

The study aimed to analyze the psychometric properties of the Addiction-Like Eating Behavior Scale (AEBS) in adolescents from Peru.

**Methodology:**

The study was psychometric in nature, conducted with a sample of 1,249 adolescents from coastal (Chimbote, *n* = 841) and jungle (Tarapoto, *n* = 408) cities in Peru. An exploratory factor analysis (EFA) was performed, and a scree plot was used to determine the factors. Additionally, a confirmatory factor analysis (CFA) was conducted to evaluate the goodness-of-fit indices used for structural validity, and the reliability of the Peruvian version of the AEBS was determined using McDonald's Omega.

**Results:**

The EFA reported the presence of three factors (KMO = 0.80 and *p* < 0.000). Furthermore, the CFA reported adequate fit indices (χ^2^ = 881.401; CFI = 0.957; TLI = 0.946; RMSEA = 0.074; and SRMR = 0.066) confirming the structure. The reliability was acceptable, with ω > 0.65 for all factors.

**Conclusion:**

The adapted version of the AEBS is valid and reliable for use with Peruvian adolescents.

## 1 Introduction

Obesity and overweight are global public health problems characterized by excessive body fat (World Health Organization, 2019). Annually, more than 4 million people die due to causes related to these issues, and there is a progressive increase in obesity among children and adolescents in urban areas of low-and middle-income countries (Poskitt, 2014). In fact, in 2022, 43% of adults worldwide were overweight and 16% were obese. The prevalence varied by region, ranging from 31% in Southeast Asia to 67% in the Americas. Additionally, 390 million children and adolescents were overweight (World Health Organization, 2024). And in Latin America, of those aged 5–19, 33.6% of children and adolescents were overweight or obese (Pan American Health Organization, 2024).

The primary factor driving the increase in overweight and obesity is diet (World Health Organization, 2024). Some studies have suggested that consuming foods high in fats and sugars may lead to eating behaviors similar to addictive conduct (Gearhardt et al., 2009). The lack of control and excessive consumption of these foods characterizes individuals with these issues, despite compromising and harming their health (Hauck et al., 2020). Moreover, the inherent characteristics of adolescents, such as low inhibitory control and impulsivity, increase the risk of developing addictive behaviors toward food (Cohen-Gilbert and Thomas, 2013; Stautz and Cooper, 2013).

A global review reports that food addiction behaviors have a prevalence of 15% in children and adolescents and 19% in overweight or obese youth (Yekaninejad et al., 2021). In Latin America, the prevalence is 35% in the clinical population and 15% in the non-clinical population (de Melo Barros et al., 2023). Additionally, this issue is more frequently observed in adolescents with mental health problems (Skinner et al., 2021). For example, a study with Chinese adolescents found that those with depressive symptoms or low self-esteem were three times more likely to exhibit addictive eating behaviors (Zhao et al., 2018). Other studies have reported higher scores in individuals with psychotic disorders, affective disorders, and addictive disorders (Horsager et al., 2022; Piccinni et al., 2021).

Several or similar instruments are used to assess addictive eating behaviors in children and adolescents (Cavicchiolo et al., 2022). For example, the Yale Food Addiction Scale for Children 2.0 (Schiestl and Gearhardt, 2018), Binge Eating Scale (BES), Palatable Eating Motives Scale (PEMS), The Eating Disorder Examination Questionnaire (EDE-Q; Berg et al., 2012), Tempest Self-Regulation of Eating Questionnaire (TESQ-E; Diotaiuti et al., 2022) and the Addiction-like Eating Behavior Scale (AEBS; Ruddock et al., 2017). Unlike other scales, such as the YFAS, which strictly adhere to substance addiction criteria to assess food addiction (Schiestl and Gearhardt, 2018), the AEBS evaluates the behavioral characteristics present in food addiction and is composed of two factors (appetitive drive/dietary control; Ruddock et al., 2017). Additionally, the AEBS has demonstrated psychometric consistency and adaptations in various countries, including France (Legendre and Bégin, 2021), Italy (Rossi et al., 2023), Turkey (Demir et al., 2021), and Brazil (Cardoso et al., 2020).

In Peru, according to the National Demographic and Family Health Survey (ENDES), the prevalence of obesity and overweight among adolescents is 67.2%, with a higher proportion in urban women (66.2%) and rural women (48.7%; Instituto Nacional de Estadística E Informática, 2021). Consequently, various social programs such as “glass of milk,” “Soup kitchens,” and “Qali Warma” have been implemented to improve nutrition in this population (Diez-Canseco and Saavedra-Garcia, 2017). However, some studies have indicated that these programs may increase the risk of overweight or obesity (Corvalán et al., 2008). In fact, more than half of the educational institutions for children and adolescents in Peru have unhealthy kiosks, facilitating access to foods that promote overweight (Arhuis-Inca and Bazalar-Palacios, 2019).

In Peru, only one study reports a prevalence of food addiction in over 34% of university students (Lopez-Lopez et al., 2021). However, this study was conducted with adults and does not provide evidence of validity and reliability for the instrument used. In this regard, the lack of evidence on the structure and reliability limits the use of the instrument in different cultural contexts and populations, such as those in the coastal and jungle regions of Peru, which are characterized by distinct geographical, economic, and dietary conditions (Matta, 2021; Turner and Klaus, 2020). Consequently, interpretations based on these evaluations could be inaccurate, as the functionality of the items and the reliability of their assessment might be assumed without proper validation (American Educational Research Association, 2014).

In response to this, the aim of this study was to evaluate the psychometric properties of the Addiction-like Eating Behavior Scale (AEBS) in adolescents in Peru. The specific objectives were: (1) Exploratory factor analysis, (2) Confirmatory analysis of the scale's structure, and (3) Reliability of the scale.

## 2 Methods

### 2.1 Design

The study was psychometric in nature, aimed at determining the psychometric properties of the Addiction-like Eating Behavior Scale (AEBS) in Peruvian adolescents. An exploratory factor analysis (EFA) and a confirmatory factor analysis (CFA) were conducted to determine construct validity, while internal consistency was estimated to assess reliability.

### 2.2 Population and sample

The population consisted of 1,249 secondary school students from private educational institutions in the coastal cities (Chimbote, *n* = 841) and jungle cities (Tarapoto, *n* = 408) in Peru. The selected cities represent two distinct cultural and socioeconomic contexts in Peru. Chimbote, located on the northern coast, has an economy centered on fishing, while Tarapoto, located in the eastern Amazon region, has socioeconomic characteristics centered on commerce, agriculture, and tourism, both cities allow for a relevant comparative analysis. Non-probability purposive sampling was used due to the availability and willingness of the educational institutions to participate in the study. Although this type of sampling limits the possibility of participation of all adolescents under the same conditions since the participants were not randomly selected, it did allow access to a broad sample from the selected educational institutions. Public educational institutions were contacted directly and selected based on their accessibility and institutional willingness to participate. Once institutional authorization was obtained, students between 12 and 17 years of age, of both sexes, were invited to participate. The inclusion criteria were: being a high school student, being within the specified age range and having the consent of the parents or legal guardians, as well as the consent of the adolescent. Exclusion criteria included: failure to provide informed consent or assent and absence during data collection, the presence of any neurodivergent condition by self-report.

### 2.3 Procedures

The study followed the guidelines of international standards proposed for test validation and adaptation (American Educational Research Association, 2014).

First, permission was requested via email from the original creators of the instrument, Dr. Ruddock and colleagues, for the validation and translation of the AEBS. The translation of the AEBS into Spanish was performed by a bilingual specialist familiar with psychological terminology, followed by back-translation by an independent translator. Subsequently, a panel of experts composed of three professionals in adolescent clinical psychology and one in psychometrics evaluated the content validity of the adapted scale, focusing on clarity, relevance, and cultural coherence for Peruvian adolescents. During this process, special attention was paid to ensure that the items were culturally appropriate, considering common eating habits, food availability, and expressions common to adolescents in Peru. Minor modifications were made to ensure that the language was understandable and adapted to the adolescents' experiences. The new adaptation sought to preserve the original meaning of the items and, at the same time, improve their cultural relevance.

Similarly, permission was requested from the director of each institution. In addition, informed consent was obtained from the parents or legal guardians of the adolescents, and assent was also obtained from the adolescents themselves prior to participation in the study. Two undergraduate psychology students, who had been trained in the use of the instrument, assisted in the classrooms during academic hours to evaluate the participants. Data collection took place over 2 weeks in August and September 2023. The data were digitized, and the principal investigator protected the data in password-protected Excel files, accessible only to the study researchers.

### 2.4 Instruments

To evaluate addictive eating behavior, the Addiction-like Eating Behavior Scale (AEBS), originally developed by Helen Ruddock and colleagues in the U.S. (Ruddock et al., 2017), was used. This instrument consists of 15 items and has two dimensions: (1) appetitive drive and (2) low dietary control. It is a five-point Likert scale (1 = never to 5 = always), with a maximum score of 75. The AEBS has demonstrated good internal consistency, with a Cronbach's alpha >0.8 for both factors (Ruddock et al., 2017).

### 2.5 Analysis plan

For the descriptive analysis of sociodemographic variables, univariate statistics such as the mean were used for age, and frequency and percentage analyses were used for sex and region. Additionally, for item analysis, the mean and standard deviation were evaluated. To assess normality, skewness, and kurtosis were used, with a range of +2.5 to −2.5 considered normal (Blanca et al., 2013).

**Figure 1:**
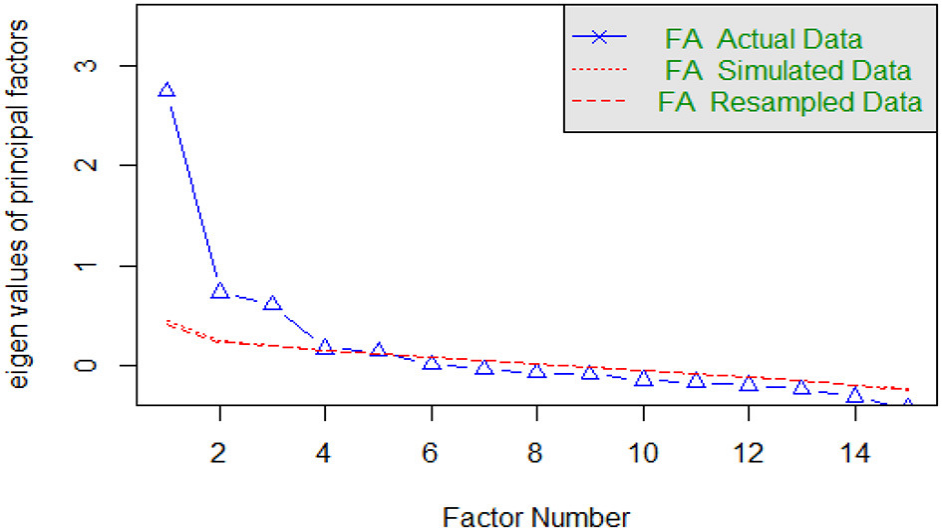
Parallel analysis scree plots.

For the factor structure analysis, the first sample was divided into 513 participants for Exploratory Factor Analysis (EFA), while the second subsample (*n* = 736) was used for Confirmatory Factor Analysis (CFA). This division ensured that the EFA and CFA were performed on separate data sets, minimizing bias and improving the validity of the model's cross-validation (Lloret-Segura et al., 2014). The EFA used the Kaiser-Meyer-Olkin (KMO) test and Bartlett's test of sphericity, with values considered adequate when KMO > 0.6 and *p* < 0.05, respectively (Hair, 2009). A minimum factor loading criterion of 0.30 was considered for item retention. Items that did not meet this threshold were eliminated from the model. This decision was made to ensure the psychometric quality and internal consistency of the adapted scale (Hair, 2009). The factor extraction method was Maximum Likelihood, recommended for large samples, and Oblimin rotation was applied due to the correlation between factors. Additionally, factor retention decisions were based on multiple criteria: visual inspection of the scree plot complemented by parallel analysis to ensure a robust method for factor determination (see Figure 1; Table 1; Fabrigar et al., 1999).

**Table 1:** Characteristics of the participants.

**Characteristics**	**AFE (*****n*** = **513)**		**AFC (*****n*** = **736)**	
	**M**	**DE**	**M**	**DE**
**Age**	13.94	1.51	14.1	1.42
	*n*	%	N	%
**Sex**			
Male	261	50.9	378	51.4
Female	252	49.1	358	48.6
**Region**			
Jungle	168	32.7	240	32.6
Coast	345	67.3	496	67.4

M, mean; SD, standard deviation.

In the present study, we used the robust Unweighted Least Squares (ULS) estimator to perform Confirmatory Factor Analysis (CFA). We decided to use the robust ULS estimator because ULS has been shown to perform adequately in the presence of ordinal data, especially when the sample size is large and the items are measured using Likert-type scales with five or more response categories (Beauducel and Herzberg, 2006). Furthermore, robust ULS is a method that does not assume multivariate normality, making it suitable for handling non-normal ordinal data, as is often the case in our study (Forero et al., 2009). In this sense, robust ULS is considered an acceptable alternative, especially when the model is correctly specified and the sample size is sufficient to produce stable estimates (Li, 2016). The model fit was evaluated through the following indices: Comparative Fit Index (CFI; >0.90), Tucker-Lewis Index (TLI; >0.95), Root Mean Square Error of Approximation (RMSEA; <0.08), and Standardized Root Mean Square Residual (SRMR; <0.06; Brown, 2015).

Reliability was determined by estimating internal consistency, as evidenced by the omega coefficient. Values greater than ω > 0.65 were considered acceptable (Hayes and Coutts, 2020). The analyses were conducted using the statistical software R Studio, JASP, and Jamovi.

### 2.6 Ethics

The study was reviewed and approved by the Research Ethics Committee of the Universidad Católica Los Ángeles de Chimbote (approval code: RESOLUTION No. 1378-2023-CU-ULADECH Católica). The ethical review of the higher institution ensured that the research complied with national regulations for research with minors. In addition, all procedures were carried out in accordance with the ethical standards established in the Declaration of Helsinki (Luis Manzini, 2000). Informed consent was obtained from the parents or legal guardians of the participants, as well as informed assent from the adolescents prior to their participation. Data confidentiality and anonymity were guaranteed.

## 3 Results

### 3.1 Descriptive analysis of the items

Table 2 shows that item 11 (“I tend not to buy processed foods that are high in fat, salt, and sugar”) had the highest average score, while item 9 (“I keep eating until I feel unwell”) had the lowest average score. Additionally, except for item 9, all other items had skewness and kurtosis values within acceptable ranges (Skewness <±2.5; Kurtosis <±2.5). The factor loadings ranged from 0.396 to 0.671, indicating an adequate relationship. Items 6 (“I can easily choose healthy foods”) and 15 (“I feel that I cannot control my weight”) were eliminated from the final analysis because they did not show acceptable factor loadings on any of the factors (values below 0.30) and their inclusion reduced the internal consistency of the model. Therefore, it was decided to exclude them to improve the overall fit of the scale.

**Table 2:** Descriptive analysis and factor loadings of items.

**Item**	***M***	***DE***	***g*1**	***g*2**	**Factor 1**	**Factor 2**	**Factor 3**
Ítem 1	2.19	1.036	0.595	−0.127	0.522	–	–
Ítem 2	2.07	1.050	0.823	0.119	0.671	–	–
Ítem 3	1.93	1.030	0.959	0.304	0.456	–	–
Ítem 4	1.54	0.869	1.681	2.466	0.615	–	–
Ítem 5	1.71	0.922	1.282	1.196	0.579	–	–
Ítem 6	2.72	1.259	0.261	−0.890	–	–	–
Ítem 7	2.35	1.201	0.590	−0.558	0.434	–	–
Ítem 8	2.39	1.028	0.473	−0.227	–	0.572	–
Ítem 9	1.41	0.854	2.334	5.196	–	0.396	–
Ítem 10	2.17	1.095	0.671	−0.308	–	0.556	–
Ítem 11	3.22	1.147	−0.045	−0.709	–	–	0.546
Ítem 12	2.96	1.121	0.205	−0.727	–	–	0.660
Ítem 13	2.75	1.087	0.318	−0.428	–	0.548	–
Ítem 14	2.90	1.185	0.192	−0.837	–	–	0.444
Ítem 15	2.35	1.204	0.583	−0.577	–	–	–

M, Mean; DE, Standard Deviation; g1, Skewness; g2, Kurtosis.

### 3.2 Exploratory factor analysis of the AEBS

The factorial solution in the EFA reveals the presence of three latent factors for the Peruvian version of the AEBS. Additionally, this model, estimated by Maximum Likelihood with Oblimin rotation, reported a KMO of 0.80 and Bartlett's test with *p* < 0.000. The sedimentation graph supports the appropriate factorial solution with its three factors (Figure 1).

### 3.3 Confirmatory factor analysis and reliability

**Figure 2:**
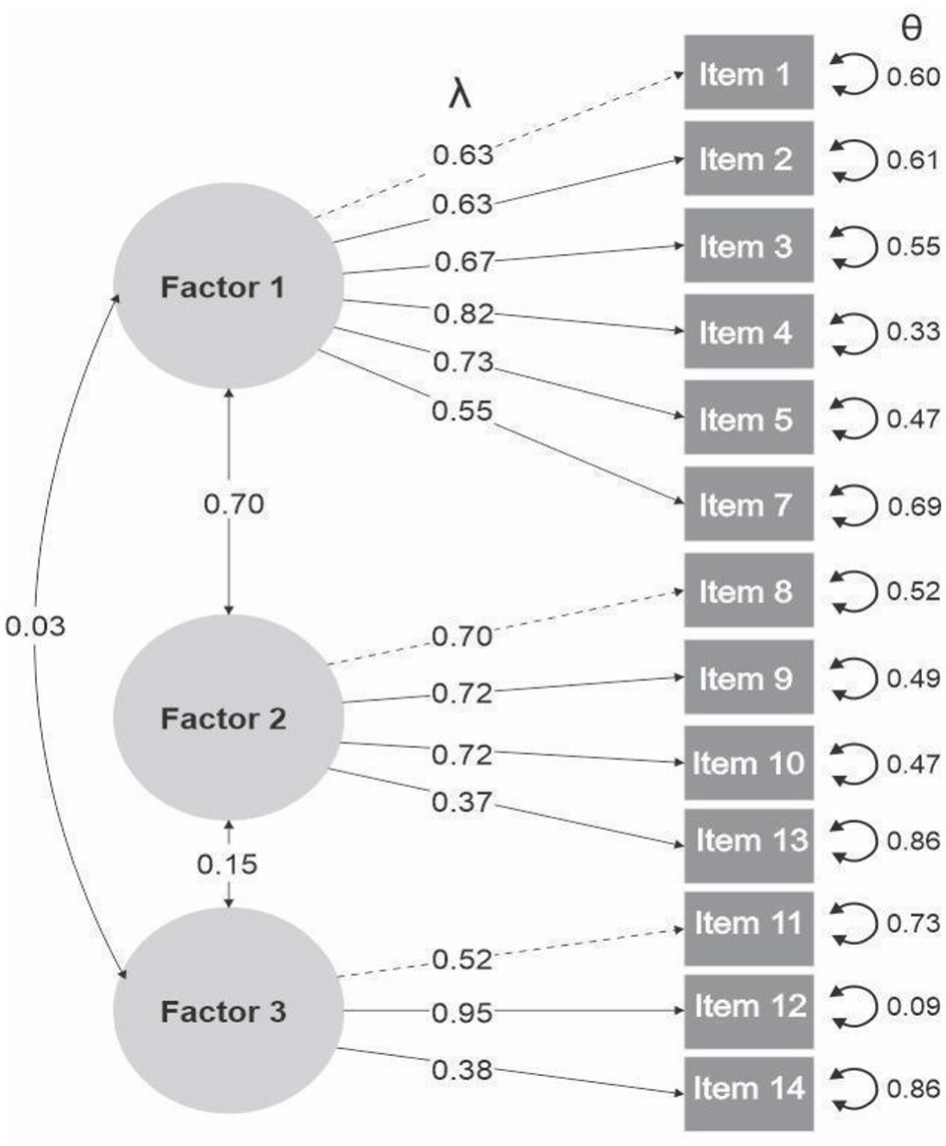
Structural equation modeling (SEM) diagram.

The evidence of structural validity for the AEBS (Table 3) reveals that the best fit corresponds to a model with three factors, reporting the following values in the total sample: χ^2^ = 881.401; CFI = 0.957; TLI = 0.946; RMSEA = 0.074; and SRMR = 0.066. Additionally, the reliability of the AEBS shows omega (ω) values >0.65 for all factors. Figure 2 illustrates that the standardized factor loadings (λ) were adequate, ranging from 0.37 to 0.95. The lowest loading was observed in Factor 2 (Item 13: “I think I have a healthy diet”), while the highest was found in Factor 3 (Item 12: “I do not consume many foods rich in fats and sugars”). The residual variances (θ) ranged from 0.09 to 0.86. In addition, the interfactor correlations were 0.70 between Factors 1 and 2, 0.15 between Factors 2 and 3, and 0.03 between Factors 1 and 3.

**Table 3:** Goodness of fit indices and reliability of the AEBS.

**Model**	**Goodness of fit indices**	
3 factors	*X*^2^(76)	881.404
	CFI	0.957
	TLI	0.946
	SRMR	0.066
	RMSEA	0.074
	Factor 1 (ω)	0.783 [0.75–0.79]
	Factor 2 (ω)	0.66 [0.63–0.69]
	Factor 3 (ω)	0.65 [0.63–0.66]

X^2^(df) for model vs. baseline. CFI, Comparative fit index; TLI, Tucker-Lewis index; SRMR, Standardized root mean squared residual; RMSEA, Root mean squared error of approximation; ω, Omega de McDonald.

## 4 Discussion

The purpose of this study was to evaluate the psychometric properties of the Addiction-like Eating Behavior Scale (AEBS) in a sample of 1,249 adolescents residing in the coastal, and jungle regions of Peru. The Peruvian version of the AEBS demonstrated adequate psychometric properties with a three-factor model and appropriate factor loadings. Additionally, internal consistency revealed good reliability of the instrument.

In our study, the Peruvian validation of the AEBS has a factorial solution composed of three factors, with each item showing a strong positive correlation with its respective factor, providing evidence of the construct validity of the AEBS. Reviewing the CFA data confirms that the factorial structure is distinct from its original two-factor version (Ruddock et al., 2017). The differences between the original two-factor model and the three-factor model found in this study highlight the importance of considering cultural context, population characteristics, and factor breakdown (American Educational Research Association, 2014). For example, in Peru, dietary practices and perceptions of food may have led to a more nuanced differentiation of eating behaviors (Gassmann et al., 2022; Perez-Leon et al., 2018). For example, in the Peruvian population, overeating is often accepted and perceived as a sign of good health (Carrillo-Larco et al., 2017). Similarly, the consumption of high-sodium, fried, and sugary foods is prevalent due to their low cost and accessibility (Leonard and Thomas, 1988; Pesantes et al., 2017). Furthermore, adolescents are in a developmental stage where they are forming their eating habits and may experience various social and emotional pressures that influence their eating behavior (Bittar and Soares, 2020; Moore, 1972; Stockebrand Gómez, 2019). Similarly, it is possible that the third factor identified in our study represents a subdivision of the original construct proposed in the AEBS. According to Ruddock et al. (2017), the original scale is composed of factors related to appetitive drives and loss of control over food intake, key aspects of addiction-like eating behaviors. In our study, the “appetitive drive” factor could have been segmented into more specific components, reflecting distinct characteristics of eating behavior in Peruvian adolescents. In fact, the third factor, composed of items 11, 12, and 14, includes elements associated with dietary restraint and the tendency to avoid unhealthy foods. In this sense, this factor can be interpreted as a form of inhibitory control over food intake, which aligns with the original theoretical foundations of the AEBS model.

### 4.1 Internal consistency

The internal consistency of the AEBS, measured by Cronbach's alpha, demonstrated acceptable reliability for the three-factor structure. This finding is consistent with the study reported with young people in Brazil, who found that the AEBS has a reliability coefficient >0.8 in all its dimensions (21). Similarly, the Italian version of the AEBS, composed of two factors (dietary control and appetite management) with 15 items, showed a McDonald's omega reliability >0.9 (Rossi et al., 2023). Additionally, the Chinese adult version of the AEBS revealed high reliability, with a total AEBS score of (α = 0.86; Ling et al., 2022).

Although two of the dimensions yielded omega coefficients of 0.66 and 0.65, it is essential to recognize that the commonly cited threshold of 0.70 for internal consistency is not a rigid or universally applicable standard. As noted by Dunn et al. (2014), the 0.70 cutoff has become widespread in the literature, yet it should not be treated as a strict rule for establishing reliability. The acceptability of a reliability coefficient must be interpreted in light of the instrument's characteristics, including the number of items, the complexity of the construct, and the contextual properties of the sample. Supporting this, Katz (2006) contends that omega values equal to or >0.65 may be considered acceptable, particularly in exploratory studies or when measuring latent variables that are inherently multifaceted. Moreover, from a clinical and applied perspective, Shrout (1998) offers a qualitative classification in which coefficients between 0.61 and 0.80 are considered to indicate moderate reliability, and those between 0.81 and 1.00 as substantial. Therefore, the reliability of the instrument can be considered adequate, especially given that the confidence interval approaches the 0.70 gold standard. This level of internal consistency supports the instrument's potential for use in clinical settings, where moderate reliability may still provide valuable information to guide assessment, diagnosis, and intervention planning.

### 4.2 Implementation of findings in the study of eating disorders

The implementation of an instrument to assess eating disorders is not only psychometrically relevant but also provides a theoretical framework consistent with recent empirical findings. Evidence indicates that binge eating and other maladaptive eating behaviors are associated with self-regulation failures, particularly in emotionally charged or stressful contexts. Individuals with bulimia nervosa have been shown to exhibit reduced proactive inhibition and dysfunctional prefrontal responses, suggesting impairments in anticipatory behavioral control (Westwater et al., 2021). Furthermore, impulsivity, especially when triggered by negative affect, has been identified as a key mediating factor between emotional distress and binge eating symptoms (Barrios et al., 2021; Wilson et al., 2021). These patterns align with the Revised Reinforcement Sensitivity Theory (rRST), which posits that heightened sensitivity of the Behavioral Inhibition System (BIS) and deficient regulation of the Behavioral Approach System (BAS) contribute to maladaptive coping strategies such as compulsive eating (Wilson et al., 2019). Additionally, individuals with eating disorders often demonstrate elevated reward and punishment sensitivity, increasing their emotional reactivity and making them more likely to use food as an emotion regulation strategy (Stapleton and Whitehead, 2014; Barrios et al., 2021). Therefore, a validated instrument enables the identification of these dynamics in adolescent populations, a group that remains theoretically and empirically understudied, while also informing targeted interventions grounded in robust psychological models.

### 4.3 Implications

Confirming the psychometric properties of the AEBS in a Peruvian context has important practical implications. It can be used as a screening tool to detect psychological distress linked to disordered eating behaviors in adolescents, which may indicate psychosocial vulnerability. As part of routine evaluations or mental health check-ups in schools, it can help psychology departments identify students at risk and refer them for timely support, potentially preventing academic problems associated with eating behavior issues. When applied at a larger scale, the instrument enables the identification of behavioral patterns across the student population, allowing schools to implement preventive interventions such as targeted workshops or structured psychoeducational programs. In clinical contexts, the instrument can assist health professionals in supporting clinical judgments during psychological or psychiatric assessments. It provides evidence on whether disordered eating is a central issue requiring intervention or an early warning sign of emerging pathology. Its use supports clinical decision-making and prioritization of treatment needs. In research, the instrument fills a gap in the limited availability of validated tools for assessing eating disorders in adolescents. It offers a standardized measure that can be used in future studies to improve understanding, tracking, and treatment of these conditions in this population.

In terms of public health, the scores obtained in the AEBS can serve as key indicators to assess the prevalence of problematic eating behaviors in adolescent populations. This can guide the design of public policies focused on promoting healthy eating habits and preventing eating disorders. For example, local health authorities could use these data to identify risk groups and design prevention programs aimed at adolescents. We further believe that by responsibly studying adolescents with respect for their wellbeing and privacy, it is useful to contribute to public health initiatives aimed at understanding and addressing adolescent eating and related problems. These types of studies serve to inform the development of specific prevention and intervention strategies that can improve adolescent health outcomes and reduce problem burden.

Furthermore, the availability of a valid and reliable scale can influence the formulation of governmental and educational strategies aimed at integrating nutritional education into the school curriculum, regulating advertising of ultra-processed foods aimed at minors, and promoting healthy food environments in schools and communities. Furthermore, our findings justify the need to expand resources in mental health and nutrition programs that strengthen care in health centers and promote the training of professionals in the identification and management of these behaviors.

### 4.4 Strengths and limitations

This is the first study worldwide that provides scientific evidence on the validity and reliability of the AEBS instrument in adolescents. Additionally, the study provides evidence for its application in adolescents from the coastal and jungle regions of Peru, offering representativeness and accuracy in the use of the Peruvian version of the AEBS. However, there are limitations to consider. First, the sample was selected by non-probabilistic purposive sampling and was limited to adolescents from two specific Peruvian cities in the northern coast and eastern jungle of Peru, which restricts the generalization of the results to populations with different sociodemographic characteristics. In that sense, the type of sampling applied in this study could have influenced the factor structure reported, since the response patterns analyzed could reflect unique characteristics of the selected sample rather than universal dimensions of the construct. Therefore, future studies should utilize stratified and probability sampling methods to better represent the diversity of the Peruvian adolescent population and examine the performance of the AEBS in different cultural contexts. Second, the study had a cross-sectional design that reduces the possibility of generating causal inferences between eating behaviors and related factors. Third, because the data were collected by self-report, there are some response biases, such as social desirability or restricted information. These limitations suggest that the following studies should replicate the findings with the application of random sampling and larger sample sizes, as well as longitudinal designs to assess the temporal stability of the AEBS. Furthermore, analyses of measurement invariance between demographic groups (e.g., sex, region, age) are needed to confirm the equivalence of AEBS between these population groups. Similarly, it is suggested to explore the associations of the AEBS scores with other psychological constructs that allow us to argue the theoretical framework that supports the scale and its criterion validity. In this sense, the findings of the present study should be interpreted with caution, and it is recommended that future studies address these methodological limitations. Finally, a potential limitation arises from the use of the ULS estimator in the CFA, rather than other estimators. In our study, we detail and support the use of the robust ULS estimator, but we also acknowledge that other estimators, such as the WLSMV, could offer additional advantages in terms of fit and ordinality management. Therefore, we suggest that future studies replicate these analyses using different estimators, such as the WLSMV, or other robust alternatives to validate the scale's factor structure.

### 4.5 Conclusions

The Peruvian version of the AEBS for adolescents has confirmed a valid and reliable structure in its three-factor model, composed of 13 items. The Peruvian version of the AEBS is a brief and easy-to-administer tool for assessing addiction-like eating behaviors. We also invite the scientific community to collaborate in further exploring the validity of the AEBS in diverse populations.

## Data Availability

The raw data supporting the conclusions of this article will be made available by the authors, without undue reservation.
