# Supplementary material for: Reliability and validity of the Addiction-Like eating behavior scale (AEBS) in a Peruvian adolescent population
Source: Front Psychol. 2025 Jun 18;16:1490893. doi: 10.3389/fpsyg.2025.1490893 (PMC12217219; doi:10.3389/fpsyg.2025.1490893)
Supplement: Supplementary file 1 [file Supplementary_file_1.docx]

Supplementary Material

**The final instrument of Food Addiction Behavior Scale (AEBS) (spanish version)**

| Responda con sinceridad marcando una X en una de las alternativas de respuestas de cada pregunta | | | | | |
| --- | --- | --- | --- | --- | --- |
|  | **Nunca** | **Rara vez** | **A veces** | **La mayoría de veces** | **Siempre** |
| 1. Sigo comiendo a pesar de sentirme lleno(a) |  |  |  |  |  |
| 2. Me sirvo porciones excesivamente grandes de comida |  |  |  |  |  |
| 3. Me resulta difícil limitar lo que como |  |  |  |  |  |
| 4. Como en exceso de manera compulsiva |  |  |  |  |  |
| 5. En cuanto a la comida, tiendo a exagerar |  |  |  |  |  |
| 6. Una vez que empiezo a comer ciertos alimentos, no puedo parar hasta acabarlos |  |  |  |  |  |
| 7. A pesar de intentar comer saludable, termino comiendo alimentos "poco saludables" |  |  |  |  |  |
| 8. Sigo comiendo hasta sentirme mal |  |  |  |  |  |
| 9. Sigo comiendo ciertos alimentos poco saludables a pesar de ser consciente de sus efectos en mi salud |  |  |  |  |  |
|  | **Totalmente en desacuerdo** | **En desacuerdo** | **Ni de acuerdo/ ni en desacuerdo** | **De acuerdo** | **Totalmente de acuerdo** |
| 10. Tiendo a no comprar alimentos procesados que sean altos en grasas, sal y azúcar |  |  |  |  |  |
| 11. No como muchos alimentos altos en grasas y azúcares |  |  |  |  |  |
| 12. Creo que tengo una dieta saludable |  |  |  |  |  |
| 13. Tiendo a no comer demasiado |  |  |  |  |  |
